# Comparison treatment of medium-sized volar fingertips defects with modified triangular neurovascular unilateral advancement flap versus digital artery dorsal perforator flap

**DOI:** 10.1186/s13018-024-04608-z

**Published:** 2024-02-03

**Authors:** Lixi Zhu, Feiya Zhou, Xian Zhang, Xue Zhang, Pinghu Jin

**Affiliations:** 1 Department of Operation Care Unit, The Second Affiliated Hospital and Yuying Children’s Hospital of Wenzhou Medical University, Wenzhou, China; 2 Department of Orthopaedics Surgery, The Second Affiliated Hospital and Yuying Children’s Hospital of Wenzhou Medical University, No. 109, XueYuan West Road, Luheng District, Wenzhou, 325000 Zhejiang Province People’s Republic of China

## Abstract

**Purpose:**

The reconstruction of medium-sized soft tissue defects of the fingertip remains a challenge for hand surgeons. The aim of this study was to compare the outcomes of modified triangular neurovascular unilateral advancement flap and digital artery dorsal perforator flap in the treatment of this injury.

**Methods:**

From May 2018 to May 2022, 70 patients with medium-sized volar soft tissue defects were enrolled. The patients were divided into two groups based on the flap type: modified triangular neurovascular unilateral advancement flap (Group A) and digital artery dorsal perforator flap (Group B). The debridement times, defect size, operation time, and flap survival rate were recorded. At follow-up, hand function, aesthetics, and complications were evaluated. Function was evaluated using the TAM score. The aesthetics of the reconstructed and donor sites were assessed using the vancouver scar scale (VSS). The static two-point discrimination of the finger pulp served as a measure of tactile agnosia.

**Results:**

A total of 10 patients were lost to follow-up for various reasons, resulting in 30 cases remaining in each group. The general information of the two groups showed no significant differences in age, sex, injury side, cause of injury, time from injury to surgery, and operation time (*P* > 0.05). Additionally, the debridement times and size of the defect were similar between the groups (*P* > 0.05). However, the operation time was significantly shorter in Group A compared to Group B (*P* = 0.001). With regard to complications, there was no significant difference between them. At one-month follow-up, TAM scores indicated that Group B performed significantly better than Group A. However, at the final follow-up period, there was no significant difference in TAM scores between the two groups. When considering the VSS, significant differences were observed between the two groups in both the reconstructed site and donor site.

**Conclusion:**

Both flaps can effectively repair medium-sized fingertip defects. Furthermore, the modified triangular neurovascular unilateral advancement flap offers anatomical reconstruction possibilities, ensuring satisfactory sensation and cosmetic contour.

## Introduction

Soft-tissue injuries to the fingertips are common and may require reconstructive surgery with a flap. It is still significantly challenging, with injuries primarily addressed at regional or community hospitals [1, 2]. Conservative treatment is a viable option for defects measuring less than 1.0 cm; however, full epithelialization can require up to 2 months, and some patients may not achieve complete healing [3]. Regarding defects sized > 1.0–1.5 cm or with bone or tendon exposure, or for patients desiring prompt wound closure, surgical intervention is frequently the preferred option. The main goal of fingertip pulp reconstruction is to restore high durability pulp, satisfactory sensibility and good appearance with minimal donor-site morbidities [4].

However, for inexperienced surgeons, choosing the optimal flap can be particularly challenging, particularly when considering the several reconstructive options and the insufficient evidence to support one flap type’s superiority over another. There are a variety of reconstructive options for the fingertip pulp loss. Local and regional hand flaps are useful for distal fingertip pulp reconstruction: retrograde flow digital artery flap [5, 6], and cross-finger flap [7] are frequently used to restore fingertip soft tissue loss. Nevertheless, they have some drawbacks, including poor sensibility and need skin graft in donor site. Free toe tissue transfer [8–10] as a reliable treatment method with good clinical outcomes in tissue similarity, motive function, and excellent sensibility. However, its complex nature limits its application in fingertip reconstruction.

The Kutler V–Y advancement flap [11] and the Atasoy advancement V–Y flap [12] are still the most widely used reconstructive methods for fingertip reconstruction. However, the application of these traditional V–Y flaps is limited by their small coverage size and advancement distance (1 cm). This study presents our experience in restoring medium-sized (1.5–2.0 cm) volar fingertip defects using a modified triangular neurovascular unilateral advancement flap and compares it with the digital artery dorsal perforator flap in terms of long-term outcomes.

## Patients and methods

### Study design

This study was approved by the Regional Ethics Committee of the Second Affiliated Hospital and Yuying Children’s Hospital of Wenzhou Medical University and adhered to the ethical guidelines outlined in the 1975 Declaration of Helsinki. All patients included in the study signed informed consent forms.

From May 2018 to May 2022, seventy patients (70 fingers) with medium-sized volar soft-tissue damage or amputated distal phalanges due to hand injury were included in this retrospective study. Based on the flap types, these patients were treated with modified triangular neurovascular unilateral advancement flap (Group A) or digital artery dorsal perforator flap (Group B). All operations were conducted in one-stage by flap coverage immediately following radical debridement. A total of 10 patients (5 in each group) were lost to follow-up for various reasons, resulting in 30 patients in each group. All procedures were performed at a single institution by the same surgical team.

### Surgical strategy

#### Group A

**Fig. 1:**
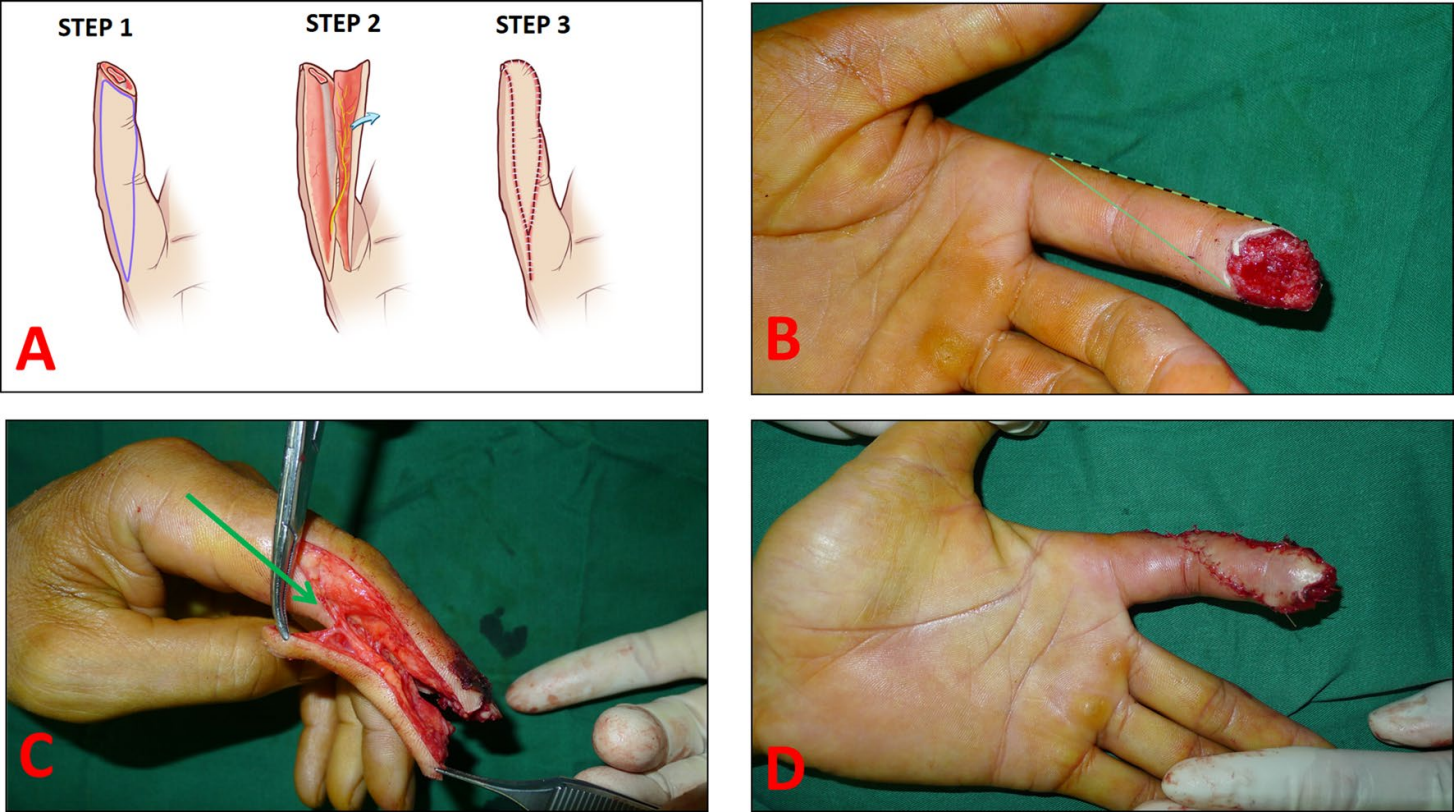
**A** A three-step schematic demonstrating that the volar incision is obliquely located in the Bruner approach and the fingertip is resurfaced after adequate advancement of the flap. **B** The flap was outlined to cover the fingertip skin loss in the left index finger of a 40-year-old man caused by a machine accident. **C** After the release of all the ligaments around the flap and raising the flap. Arrow indicates the neurovascular bundle in the proximal apex of the flap. **D** Intraoperatively instant view of fingertip loss that resurfaced with the flap

Axillary block anesthesia was administered with arm pneumatic tourniquet control before surgery. A preoperative Allen test was conducted to assess the patency of both digital arteries for each injured digit. An oblique triangle is shown in STEP1 in Fig. 1: (1) the base of the triangle is equal to the width of the wound. The lateral side of the triangular flap is the “finger lateral approach” line. (2) the volar incision of the flap is on the contralateral side of the triangle, located obliquely at the opposite margin of the defect from the proximal apex of the flap. Additionally, the proximal apex of the triangular flap was positioned at the level of the proximal phalanx and did not extend beyond the metacarpophalangeal crease (Fig. 1A, B). All fibrous septa on the oblique side were divided, and the fibros bands in the proximal apex were divided under magnifying loupes, allowing for adequate advancement of the flap (Fig. 1C). Care was taken to ensure that the neurovascular bundle was not damaged. The flap was raised and advanced obliquely to cover the fingertip defect, incorporating the neurovascular bundle within the flap. The base of the flap was sutured to the remnant nail, and the secondary defect was closed in a V–Y type closure.

#### Group B

The flap rotation point is positioned approximately 0.5 cm below the lateral margin of the dorsal transverse crease of the proximal (or distal) interphalangeal joint. This point is also where the dorsal branch of the artery exits at each joint. The axis of the flap follows the diagonal line of each joint. The distance from the exit point of the flap to the edge of the wound determines the length of the flap's rotational arc. The design of the flap's rotational arc follows a triangle shape. Due to the need for a rotational arc, the length of the flap usually needs to be greater than the length of the skin defect by approximately 1.5 cm.

All surgeries were performed under brachial plexus block anesthesia with an upper arm inflatable tourniquet. The design line of the dorsal flap was incised along the proximal, lateral, and triangular flap parts (non-effective area of the flap), with a depth of incision at the upper layer of the tendon fascia. The incision was made from the proximal end to the rotation point, and sharp dissection was performed at the upper layer of the tendon fascia. The operation was performed under a threefold magnification. Then, the skin of the two edges of the triangular flap was made to be subdermally free and pulled to expose the tissue at the base. At the rotation point, about 1 cm away, the digital artery was exposed, and it could be observed that the skin branch vessel (non-joint branch) emitted from the digital artery toward the back. After cutting it off, bipolar coagulation was used for hemostasis. The fascia flap containing about 0.5 cm wide of the digital artery dorsal branch was freed and turned over to cover the wound. If sensation needs to be reconstructed, during the process of freeing the flap, the digital nerve dorsal branch will be included in the flap. After rotation, 9-0 prolene suture under microscopic operation will be sutured with the digital nerve break end inside the wound. The donor site was covered with full-thickness skin taken from the forearm.

### Postoperative treatment

After the operation, the injury hand was asked immobilized in a functional position for approximately 3 days, which can prevent vascular pedicle pulling. The dressing was windowed to allow part of the flap to be exposed for evaluation of blood supply, while also preventing pressure on the flap after bleeding. The dressing was replaced in a timely manner. Anticoagulation therapy and routine use of antibiotics were continued for 5 days after the start of flexion and extension functional exercises. After approximately 2 weeks, the wound sutures were removed, and functional exercises were intensified to provide timely tactile stimulation of the injured fingertips. The patient then entered the recovery phase.

### Data collection and assessment parameters

Demographic data collected on the patients included age, gender, side of injury, cause of injury, and time from injury to surgery. The perioperative assessment parameters included debridement times, size of the defect, operation time, and flap survival rate. At the last follow-up, hand function and aesthetics were evaluated. Complications associated with primary surgery (necrosis, infections, and hematoma) and those occurring during follow-up were recorded. Function of the finger was evaluated using the American Society for Surgery of the Hand (ASSH) Total Active Movement (TAM) score [13] in one month follow-up and last follow-up. Total active motion was calculated as the difference between total active flexion (MCP + PIP + DIP) and total extension deficit (MCP + PIP + DIP). MCP denotes metacarpophalangeal joint, PIP proximal interphalangeal joint, and DIP distal interphalangeal joint. The grade was classified as excellent (100% normal), good (75–99% normal), fair (50–74%), or poor (< 50% normal). The aesthetics of both the reconstructed and donor sites were evaluated using the Vancouver Scar Scale (VSS) [14], which assesses pigmentation, vascularity, pliability, and height. The static two-point discrimination (2-PD) [15] of the finger pulp was employed as a measure of tactile agnosia, indicating finger nerve recovery. All tests were performed by an independent senior hand surgeon.

### Statistical analysis

The data were analyzed using Student’s *t*-test for unpaired samples and the chi-squared test. A *P* value < 0.05 was considered statistically significant. The data are presented as the mean ± standard deviation. Statistical analyses were performed using SPSS version 20.0 software.

## Results

### Patient characteristics

A total of 10 patients were lost to follow-up from the admitted patients for various reasons, resulting in 30 cases remaining in both groups. There were 20 males and 10 females in group A, while 21 males and 9 females were in group B. As shown in Table 1, no significant difference was found between the two groups in regard to the demographic characteristics such as age, sex, injury side, cause of injury, and time from injury to surgery (*P* > 0.05).

**Table 1:** Demographic date

*N*	Group A	Group B	*t* or *X*^2^	*P*
	30	30		
Age(year)	43.1 ± 14.1	41.90 ± 12.8	*T* = 0.346	*P* = 0.731
*Gender*			*X*^2^ = 0.077	*P* = 0.781
Male	20	21		
Female	10	9		
*Side of injured*			*X*^2^ = 0.067	*P* = 0.795
Right side	17	16		
Left side	13	14		
*Cause of injury*			*X*^2^ = 0.167	*P* = 0.920
Machine injury	20	21		
Sharp injury	4	3		
Burns	6	6		
*Time from injury to surgery (h)*	4.77 ± 2.0	4.97 ± 1.9	*T* = 0.396	*P* = 0.694

### Perioperative data

The results from perioperative data are summarized in Table 2. For severe contamination and severe injuries, more than one debridement operation may be required. What’s more, the debridement time and the area of the remaining wound after thorough debridement were no significant difference between the two groups (*P* > 0.05). However, the operation time of the two groups was statistically different (*P* = 0.001).

**Table 2:** Perioperative data

*N*	Group A	Group B	*T* or *X*^2^	*P*
	30	30		
Debridement times	1.1 ± 0.3	1.2 ± 0.4	*T* = 0.75	*P* = 0.456
Size of the defect (cm^2^)	1.46 ± 0.5	1.55 ± 0.5	*T* = 0.708	*P* = 0.482
Operation time (min)	33.9 ± 5.1	52.77 ± 4.9	*T* = 0.978	*P* = 0.001*

*p<0.05

### Complications, function and aesthetics outcomes

During the follow-up period, a total of 10 patients were lost to follow-up for various reasons, while the other patients in this study received at least 6 months of follow-up. During the follow-up period, there was no significant difference between the two groups in terms of complications (Table 3, *P* > 0.05). The superficial wound infection was resolved with repeated dressing and systemic antibiotics. Scar healing was achieved in all partially necrotic flaps through routine dressing.

**Table 3:** Complications, function and aesthetics of the two groups

*N*	Group A	Group B	*T* or *X*^2^	*P*
	30	30		
Follow-up period (month)	10.7 ± 2.1	9.7 ± 2.9	*T* = 1.524	*P* = 0.133
*Complications*			*X*^2^ = 2.308	*P* = 0.129
Infection	1	2		
Necrosis	1	4		
*TAM score at one month follow*			*X*^2^ = 6.933	*P* = 0.031*
Excellent	10	20		
Good	14	6		
Poor	6	4		
*TAM score at last follow*			*X*^2^ = 0.132	*P* = 0.936
Excellent	25	24		
Good	4	5		
Poor	1	1		
2-PD (mm)	4.43 ± 1.2	5.5 ± 1.8	*T* = 2.71	*P* = 0.009*
Vancouver scale (Reconstructed site)	4.13 ± 0.3	3.92 ± 0.5	*T* = 2.138	*P* = 0.037*
Vancouver scale (Donor site)	4.0 ± 0.4	3.20 ± 0.3	*T* = 9.012	*P* = 0.001*

*p<0.05

The function of the digital assessment with TAM scores at the follow-up in one month after surgery showed that there was significant difference between the two groups (Table 3, *P* < 0.05). At the last follow-up period, the TAM scores showed that there was no significant difference between the two groups (Table 3, *P* > 0.05). In VSS, there were significant differences between two groups in the reconstructed site and donor site (Table 3, *P* < 0.05). In the reconstructed site, the score in group A was higher than that in group B. In the donor site, the score was also higher in group A.

### Case reports

*Case 1*

**Fig. 2:**
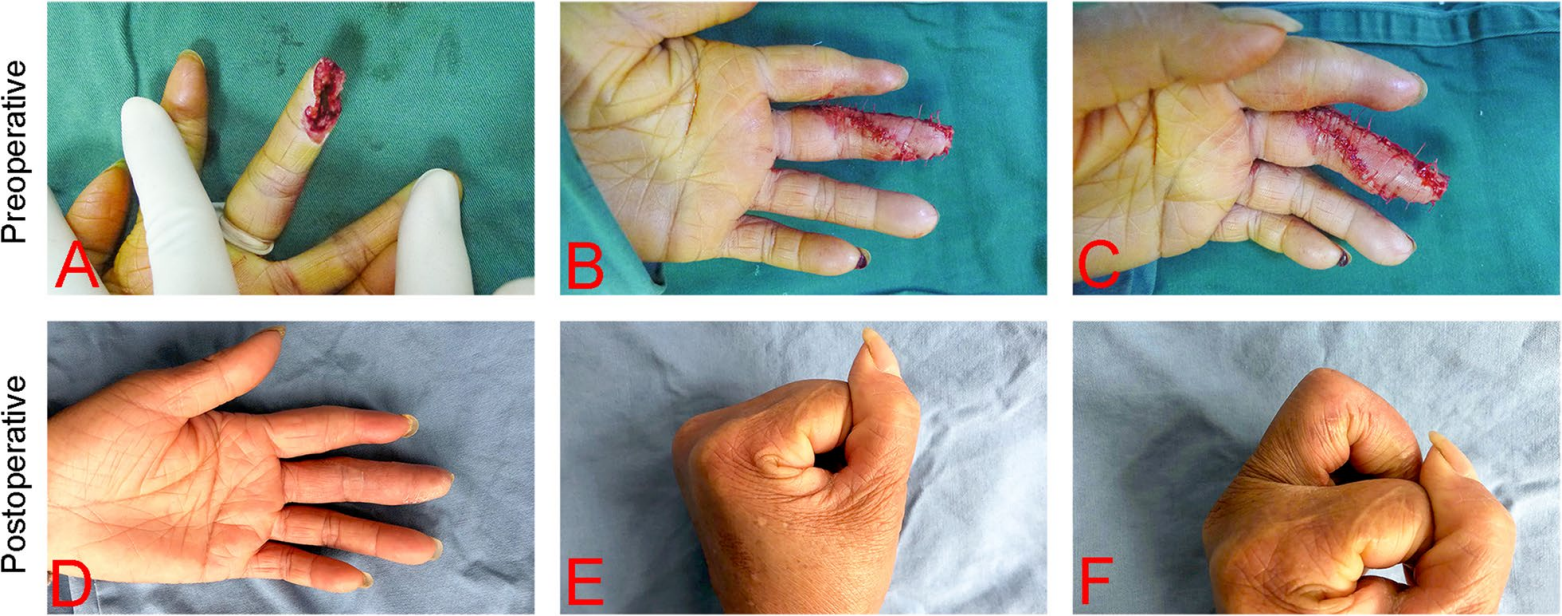
**A** and **B** A 45-year-old woman presented with a soft tissue defect on her left distal middle and ring fingers caused by a machine crush injury. **C** Resurfacing with a triangular neurovascular unilateral advancement flap. **D** and **E** One-year postoperative view. **F** Postoperative function indicating that no contracture occurred

A 45-year-old woman presented with a 2 × 1.5 cm soft tissue defect on her left distal middle finger, with flexor tendon and phalanx exposed (Fig. 2A). The injury was caused by an electric saw, and the wound defect was inclined to the ulnar side. A triangular neurovascular unilateral advancement flap was raised from the radial side of the middle finger and resurfaced to the defect wound (Fig. 2B, C), and the donor site was directly sutured. The static 2-point discrimination was tested six months after the surgery, with the result indicating that the flap was restored to 4 mm. Furthermore, the scar in the volar region did not affect motor function (Fig. 2D–F). There was no significant difference in the flexion and extension functions between the affected and the healthy sides. TAM scores show excellent at the last follow-up.

*Case 2*

**Fig. 3:**
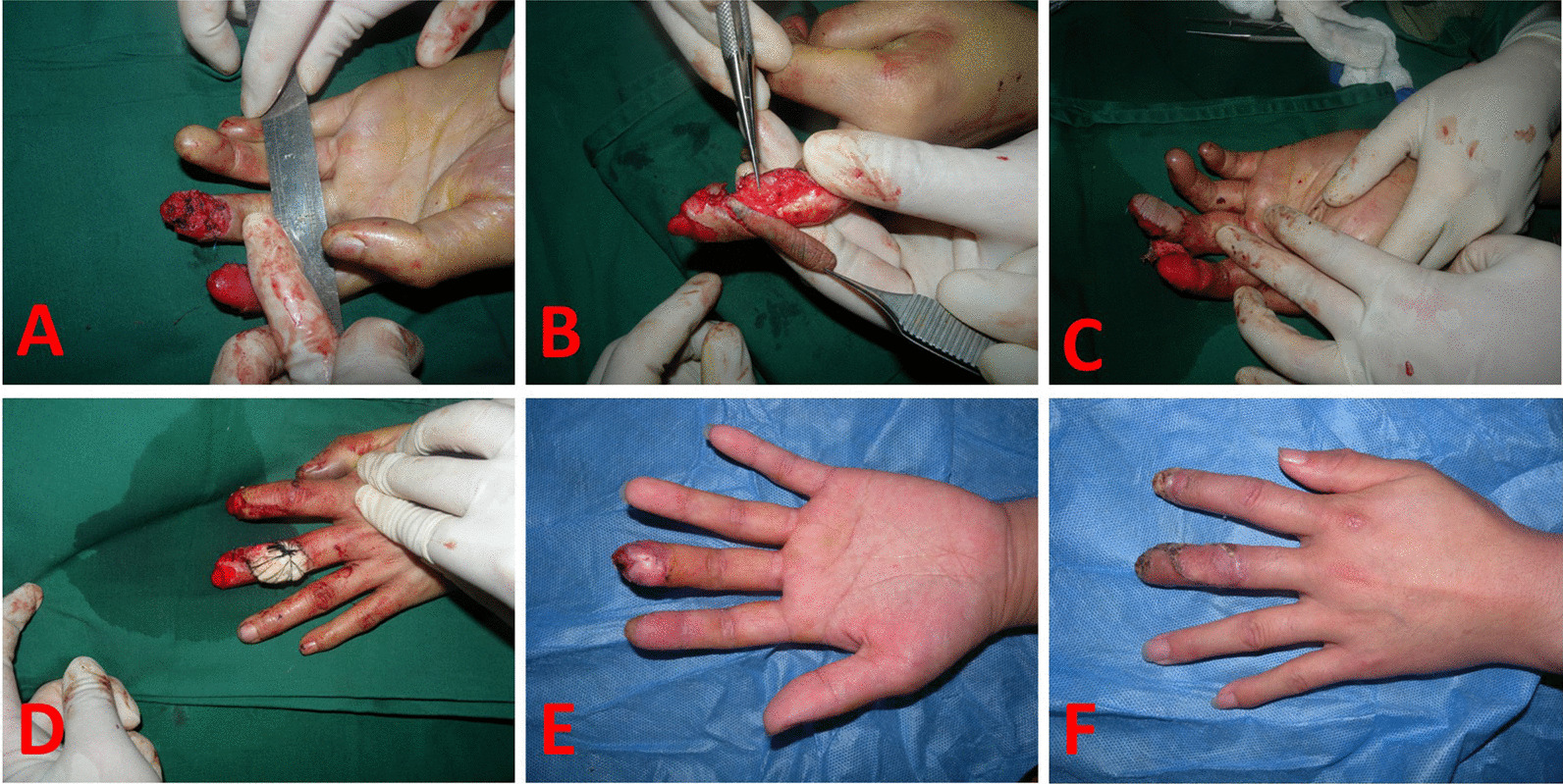
**A** A 40-year-old man presented with a soft tissue defect in his left distal finger pulp in the middle finger caused by a machine crush injury. **B**, **C** A digital artery dorsal perforator flap was raised to cover the wound. **D** The donor area was covered by skin grafting. **E**, **F** Postoperative function indicating that no contracture occurred

A 40-year-old man presented with a soft tissue defect in his left distal finger pulp in the middle finger caused by a machine crush injury (Fig. 3A). The size of the defect in the distal pulp of the middle finger was 2.5 × 1.5 cm, and the bone was exposed. A digital artery dorsal perforator flap was raised to cover the wound (Fig. 3B, C). The donor area was covered by skin grafting (Fig. 3D). The flap survived rapidly, and a nearly normal finger motive function was regained after three weeks of rehabilitation. A 2-point static discrimination was tested after one year and the result indicated that the flap was restored to 6 mm (Fig. 3E, F).

## Discussion

The fingertip pulp plays a crucial role in daily activities, such as using smartphones and tablets [14]. A volar skin defect on the fingertip is one of the most common hand injuries. Therefore, the optimal reconstruction of volar fingertip defects requires padding, maximum tactile gnosis coverage, adequate finger length, and satisfactory aesthetic contour [15]. Typically, there is agreement that replantation is the optimal treatment for fingertip loss restoration. However, several reconstructive flaps have been described with varying outcomes in cases where replantation is not feasible [16]. These methods include secondary intention healing, Kutler and Atastoy V–Y advancement flap, triangular neurovascular advancement flap, cross-finger flap, retrograde flow digital artery flap, and free toe tissue transfer [6–9, 17].

Healing by secondary intention is an effective treatment for individuals with no exposed phalanx and tendons. Nevertheless, it poses a significant risk of phalangeal osteomyelitis and tendon necrosis, and it results in significant scar formation [16]. This scar can limit the motion and sensory function that is essential for the final function of the finger. Cross-finger flap [17] has several drawbacks, including two-stage operative treatment, a relatively small tissue pad, skin grafting at the donor site, and damage to the uninjured finger. Free partial toe flap [18] transfer may perfectly meet the high demands of fingertip pulp reconstruction in a like-to-like manner. However, this procedure is time-consuming and technically challenging. Additionally, most patients struggle with postoperative management. Retrograde flow digital artery flap [19] and digital artery dorsal perforator flap [20] can cover small to medium-sized skin defects on the fingertip. These flaps are useful for fingertip pulp reconstruction. Nevertheless, skin grafting at the donor site is necessary, adding new morbidity to the uninjured finger and sacrificing one main digit artery. Common postoperative issues include venous congestion, flexion contracture, and cold intolerance [21].

The traditional V–Y advancement flaps are divided into two main types: the Kutler V–Y flap and the Atasoy flap. These flaps offer several advantages, including simple surgical procedures, minimal donor morbidity, tissue similarity, satisfactory sensory restoration, and good cosmetic contours. However, their coverage size is limited to within 1 × 1 cm, making them unsuitable for medium-sized fingertip soft tissue losses. This limitation prompted Venkataswami [22] to develop the oblique triangular flap for broader coverage, particularly for oblique lateral and medial fingertip losses. Nevertheless, even this flap cannot provide sufficient tissue for total skin pulp loss in the fingertip. This led us to modify and enhance it to achieve longer advancement and broader coverage without the need for skin grafts. In the present study, the lengths of defects treated with the modified unilateral triangular neurovascular advancement flap (alternatively called the modified V–Y flap) ranged from 1.5 to 2.0 cm. With 30 cases of patients, only one case experienced partial necrosis. Thus, this flap is a safe and reliable choice for covering the entire distal finger pulp defect. However, the indications for this modified flap are strictly limited. The wound size should be chosen to be 1.5–2.0 cm or less, as larger wounds may lead to poor coverage or local necrosis. For wounds larger than 2.0 cm, we recommend using other flaps for coverage, such as the digital artery dorsal perforator flap.

The modified unilateral advancement flap offers several advantages due to its minimal technical requirements. There is less need for artery dissection, resulting in a shorter surgical time and simplified technical demands. When raising the flap, the neurovascular pedicle is anatomically dissected at the proper digital artery trunk, while the perforator branches remain intact within the fascial tissue. This approach saves time and reduces the overall difficulty compared to the digital artery dorsal perforator flap. Additionally, the modified flap does not require anatomical tracing, identification, or separation of the digital artery. Furthermore, it does not require grafting for the donor site, further reducing surgical time. In this study, the operation time for the modified flap was 33.9 ± 5.1 min, significantly shorter than that of the digital artery dorsal perforator flap (52.77 ± 4.9 min).

The digital artery dorsal perforator flap, as described in reference [5], is a pedicled flap that receives its nourishment from the palmar proper artery of one finger. This flap is frequently used to repair soft tissue defects in the distal segment of the finger pulp. However, it requires sacrificing or freeing one side of the proper digital artery. The fingertip rotation flap [23], on the other hand, necessitates complete detachment of the flap while only preserving the vascular pedicle of the finger artery. After the flap is rotated by 90°, the donor site wound is exposed on the finger side, necessitating free skin grafting. In our surgical approach, the donor-site skin defect on the palmar side of the finger is more amenable to application of a full-thickness skin graft. Post-operative flap removal leaves behind more soft tissue in the donor area compared to the fingertip rotation flap [22], promoting graft survival and preventing significant depression upon healing.

In terms of postoperative finger function recovery as measured by TAM score, long-term follow-up showed similar outcomes with no significant differences between the two groups (*P* > 0.05). However, one month postoperatively, patients with the digital artery dorsal perforator flap exhibited superior TAM scores compared to those with the modified triangular neurovascular unilateral advancement flap. We attribute this to the larger injury scope during the harvesting process of the modified flap, necessitating incision of the entire finger body to cover the distal wound. Consequently, early healing is slower with longer recovery time. Nevertheless, as this flap preserves the integrity of the digital artery and digital nerve without causing damage, and the incisions are linear, it leads to good recovery in the later stages. Consequently, there are no differences in long-term finger function between this flap and the digital artery dorsal perforator flap.

The modified triangular neurovascular unilateral advancement flap offers several advantages: (1) It allows for maximum retention of finger length and restoration of shape, with a maximum advancement length of 2.0 cm, effectively covering finger wounds. The flap carries one side of the nerve, preserving sensation, which is crucial for daily life and work prognosis. (2) The donor and recipient areas are located within the same finger, eliminating the need for additional trauma when repairing the wound, without scarring from skin grafting or other issues. (3) The surgical operation is relatively straightforward, causing minimal donor-site damage, and the wound can be directly sutured, eliminating the need for skin grafting or secondary flap detachment. (4) It can be performed by a single surgical doctor under local anesthesia with a short operation time and no need for post-surgical fixation. Fingers can be actively moved early.

Here are some surgical precautions to consider: (1) Carefully dissect the blood vessels and nerves to avoid intraoperative damage. (2) To ensure adequate blood supply, choose patients with proximal damage to the lateral artery and nerve, and use this technique with caution. (3) The flap vascular pedicle should be fully dissected, and there should be no significant tension during suturing to prevent affecting flap blood supply. If the flap pedicle tension is high, the artery and nerve can be dissected proximally to relieve the tension. (4) The amount of finger pulp tissue and the extensibility of the finger artery are limited, so this flap is not suitable for larger wound repairs. Generally, the mobile distance should not exceed 2.0 cm. (5) Preoperatively, the need for flap vascular dissection length should be fully considered to avoid affecting surgery by applying a rubber tourniquet at the base of the finger. Some surgeries may require brachial plexus block anesthesia with upper arm or forearm tourniquet bleeding control.

## Conclusion

Both flaps can effectively repair medium-sized fingertip defects. Furthermore, the modified triangular neurovascular unilateral advancement flap offers anatomical reconstruction possibilities, ensuring satisfactory sensation and cosmetic contour.

## Data Availability

We do not wish to share our data, because some of the patients’ data regarding individual privacy, and according to the policy of our hospital, the data could not be shared to others without permission.
